# Long-term oncologic outcomes of robot-assisted pancreaticoduodenectomy versus open pancreaticoduodenectomy for pancreatic cancer

**DOI:** 10.1007/s00464-025-11833-y

**Published:** 2025-07-09

**Authors:** Younsoo Seo, Hye-Sol Jung, Youngmin Han, Inhyuck Lee, Go-Won Choi, Yoon Soo Chae, Won-Gun Yun, Young Jae Cho, Wooil Kwon, Joon Seong Park, Jin-Young Jang

**Affiliations:** Department of Surgery and Cancer Research Institute, Seoul National University College of Medicine, 28 Yongon-Dong, Jongno-Gu, Seoul, 110-744 Republic of Korea

**Keywords:** Robot-assisted pancreaticoduodenectomy, Open pancreaticoduodenectomy, Pancreatic cancer, Long-term outcomes, Adjuvant therapy

## Abstract

**Background:**

Robot-assisted pancreaticoduodenectomy (RPD) has been gaining attention for its potential benefits in short-term surgical outcomes compared with open pancreaticoduodenectomy (OPD) in pancreatic cancer. However, the evidence of its long-term oncological efficacy is limited.

**Methods:**

This retrospective study compared the long-term outcomes of RPD and OPD in patients with pancreatic cancer at Seoul National University Hospital between January 2015 and October 2023. Patients with stage III or IV disease and those who underwent open surgery were excluded. Propensity score matching (PSM) at a 1:2 ratio was performed based on sex, age, the American Society of Anesthesiologists (ASA) class, and initial resectability. The primary outcomes were overall survival (OS) and disease-free survival (DFS), and the secondary outcomes were postoperative recovery and complication rates.

**Results:**

In total, 522 patients (82 RPD and 440 OPD) were reviewed, and 82 RPD and 164 matched OPD patients were analyzed after PSM. The mean age was 64.8 years in the RPD and 65.2 years in the matched OPD group; there were 51.2% male patients in the RPD and 52.4% in the OPD groups. After PSM, patients with RPDs showed better OS and DFS (5-year OS 58.2% vs. 32.3%, P = 0.001; 5-year DFS: 44.6% vs. 24.7%, P = 0.005). The R0 resection rates and harvested lymph node (LN) number were also comparable (RPD versus OPD: R0 resection rate 92.7% versus 90.9%, P = 0.809; harvested LNs 20.0 ± 7.3 versus 23.1 ± 11.5, P = 0.090). Additionally, patients with RPD had a shorter postoperative recovery time and relatively higher adjuvant therapy completion rate, although the difference was only marginally significant (91.6% vs. 75.4%, P = 0.062).

**Conclusion:**

RPD is a feasible alternative to OPD, with potential advantages of early recovery without compromising long-term outcomes after PSM. However, further prospective studies are required to confirm these findings.

**Graphical abstract:**

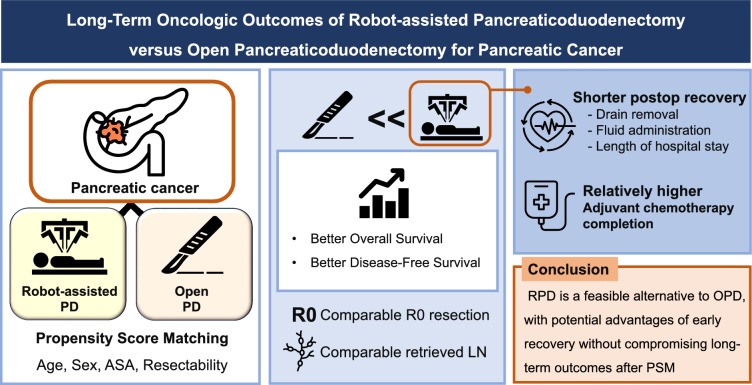

Robot-assisted surgery is an emerging field of surgery and its applicability is expanding exponentially, driven by the evolution of robotic ergonomics and instruments. A meta-analysis reported that robotic abdominopelvic surgery offers several advantages over open surgery [1]. However, the application of robotic platforms to pancreaticoduodenectomy (PD) remains restricted owing to technical challenges. Moreover, in pancreatic cancer, the presence of pancreatitis, fibrosis, and vessel invasion significantly increases the surgical complexity. Consequently, robot-assisted PD (RPD) has primarily been performed in high-volume centers [2, 3].

However, several studies have reported an increasing number of robotic pancreatic surgeries from various centers and a growing body of postoperative outcome data with favorable results compared to open methods [2, 4–15]. Additionally, with advances in neoadjuvant therapy, the demand for pancreatic cancer surgery and expectations for minimally invasive techniques are growing.

Despite the merits of robotic surgery, there is still an ongoing controversy among oncologists regarding the feasibility of minimally invasive surgery, including robotic surgery, for cancer [16]. In line with this, current studies addressing RPD outcomes often include diverse inclusion rates for pancreatic cancer, ranging from 24–81%, lacking data focused exclusively on pancreatic cancer [5, 7, 10, 11, 15].

Most studies have focused on the short-term outcomes of RPDs, primarily addressing technical and perioperative recovery. While short-term outcomes of RPD and open surgery are comparable, data on long-term effects remain limited. This study aimed to compare the long-term oncological outcomes of RPD with those of open PD (OPD) for pancreatic cancer.

## Materials and methods

### Patients and selection criteria

**Fig. 1:**
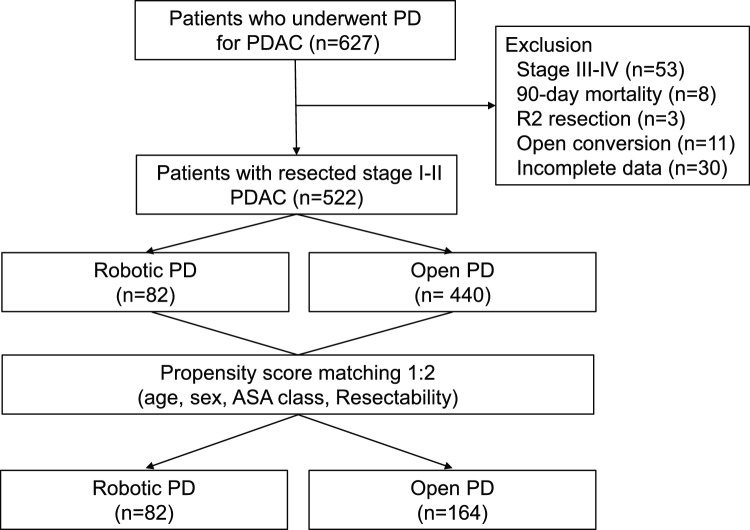
Flow chart of patient selection

All patients who were diagnosed of pancreatic ductal adenocarcinoma (PDAC) and underwent RPD or OPD at Seoul National University Hospital (SNUH), Seoul, South Korea, between January 2015 and October 2023 were retrospectively reviewed in this study. To minimize selection bias, patients with pathological stage III or IV PDAC were excluded as surgeons might be more likely to prefer open surgery for advanced cancers, potentially confounding the comparison of survival outcomes. To clarify the classification of the operation method, 11 patients who were converted to open surgery during the operation were excluded. Also, 11 patients who underwent incomplete (R2) resection (n = 3), or within 90-day mortality (n = 8) were excluded. Thirty patients with incomplete or unavailable clinical data were excluded (Fig. 1). These patients either became lost to follow-up or transferred to other hospitals immediately after surgery, making it impossible to collect reliable information on recurrence or survival outcomes. This study was approved by the Institutional Review Board, which waived the requirement for obtaining informed consent from patients (IRB No.: 2407-114-1553).

### Data collection

Clinical data were collected through a retrospective review of electronic medical records. Baseline demographics, clinical and pathological data including tumor marker levels, tumor size, clinical stage classified by initial tumor resectability (resectable, borderline resectable, locally advanced, or metastatic), and pathological stage were collected. Other oncological treatment history data, including neoadjuvant or adjuvant therapy data, were also retrieved. The primary oncological outcomes were assessed based on the overall survival (OS) and disease-free survival (DFS). OS was defined as the time from the date of surgery to the date of death from any cause, whereas DFS was defined as the time from the date of surgery to the date of the first documented recurrence or death during the follow-up period. Secondary outcomes were assessed based on perioperative and pathological outcomes, including the operation time, blood loss, number of retrieved lymph nodes, resection margin status, complication rate, and length of postoperative hospital stay. The completion rate of adjuvant therapy and the time from the date of surgery to the initiation of adjuvant therapy were also assessed. The time to first passage of flatus, removal of the surgical drain, discontinuation of fluid infusion, and removal of intravenous patient-controlled analgesia (IV PCA) were measured as postoperative functional recovery indicators.

### Surgical procedures

All RPD and OPD were performed by the same team at SNUH using the same protocol among the surgeons. The detailed protocol was described in a previously published report from the same institution [17].

### Statistical analysis

Propensity score matching (PSM) was performed at a 1:2 ratio for the RPD and OPD groups based on the sex, age, the American Society of Anesthesiologists (ASA) class, and initial resectability. Categorical variables were compared using the chi-square test or Fisher’s exact test, while continuous variables were analyzed using the Student's t-test or Mann–Whitney U test, as appropriate. Survival outcomes, including OS and DFS, were assessed using the survival analysis method with the Kaplan–Meier curve and compared using the log-rank test. Survival rates are presented with 95% confidence intervals (CI). Cox proportional hazards regression analysis was used to identify factors associated with survival, presenting hazard ratios (HR) with 95% confidence intervals (CI). Statistical significance was set at P < 0.05. All statistical analyses were performed using R software, version 4.4.1 (R Foundation for Statistical Computing).

## Results

**Fig. 2:**
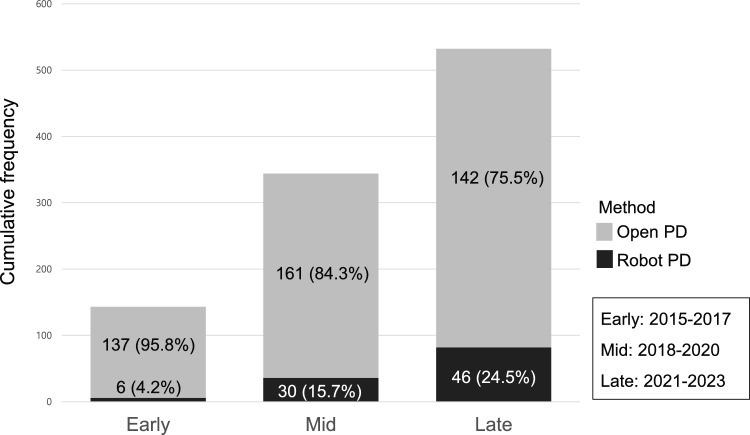
Trends in the number of RPD and OPD cases for PDAC over time in SNUH. *RPD* Robot-assisted pancreaticoduodenectomy, *OPD* open pancreaticoduodenectomy, *PDAC* Pancreatic ductal adenocarcinoma, *SNUH* Seoul National University Hospital

Of total 522 patients with PDAC who underwent either RPD or OPD, 82 underwent RPD and 440 underwent OPD. The two groups were matched in a 1:2 ratio, resulting in 82 RPD patients and 164 OPD patients for the final analysis (Fig. 1). The overall trends in the cumulative number of RPD and OPD performed over time at SNUH are shown in Fig. 2. The entire period was divided into three intervals of three years each: early period (2015–2017), mid-period (2018–2020), and late period (2021–2023). During this period, the number of RPDs performed was estimated, showing an increase in proportion from 4.2% in the early period to 24.5% in the late period.

### Baseline characteristics

The baseline characteristics of the patients who underwent RPD and OPD, both before and after PSM, are summarized in Table 1. Mean age of RPD and OPD groups were 64.8 ± 10.9 years old and 64.6 ± 9.2 years old respectively, and male patients were 51.2% of RPD group and 55.9% in OPD group before PSM. The RPD and total OPD groups showed significant differences in ASA class distribution and initial carbohydrate antigen (CA) 19–9 (U/L, median [interquartile range], RPD 58.0 [9.0–299.0] versus OPD 110.7 [19.0–619.5]). Differences between the two groups before PSM were eliminated. The initial clinical stages differed between the two groups had different proportions (RPD versus OPD (before PSM), resectable 89.0% versus 30.5%, borderline resectable 8.5% versus 41.4%, locally advanced 2.4% versus 15.5%, and metastatic 0% versus 12.7%) (P < 0.001). After PSM, the proportions of clinical stages were matched between the two groups: resectable, 89.0% versus 81.7%; borderline resectable, 8.5% versus 15.9%; locally advanced, 2.4% versus 2.4%; and metastatic, 0.0% versus 0.0% (P = 0.316). In addition, pathological TNM stages were comparable after matching (P = 0.707). The OPD group had more patients who underwent neoadjuvant therapy (RPD 14.6% versus OPD (before PSM) 48.9%; (after PSM) 32.3%) and combined vessel resection during surgery (RPD 6.1% vs. OPD (before PSM) 29.5%; (after PSM) 25.0%). The proportions of cases per time period was 56.1% for RPD and 39.0% for OPD in the period of 2021–2023, and 43.9% for RPD and 61% for OPD in the period of 2015–2020 in the matched group.

**Table 1:** Baseline demographics and clinicoathologic characteristics of RPD and OPD, before and after propensity score matching

			Before PSM		After PSM	
Variables		RPD (n = 82)	OPD (n = 440)	P-value	OPD (n = 164)	P-value
Age (years), mean ± SD		64.8 ± 10.9	64.6 ± 9.2	0.895	65.2 ± 9.6	0.958
Sex (male), n (%)		42 (51.2)	246 (55.9)	0.507	86 (52.4)	0.964
BMI (kg/m2)		23.3 ± 7.1	22.9 ± 6.4	0.617	22.2 ± 6.1	0.351
ASA class, n (%)	I	7 (8.5)	49 (11.1)	0.047*	18 (11.0)	0.317
	II	66 (80.5)	322(73.2)		122 (74.4)	
	III	9 (11.0)	69 (15.7)		24 (14.6)	
Initial CA 19–9 (U/L), median [IQR]		58.0 [9.0–299.0]	110.7 [19.0–619.5]	0.025*	89.5 [21.0–575.0]	0.083
Initial clinical stage, n (%)	R	73 (89.0)	134 (30.5)	< 0.001*	134 (81.7)	0.316
	BR	7 (8.5)	182 (41.4)		26 (15.9)	
	LA	2 (2.4)	68 (15.5)		4 (2.4)	
	M	0 (0.0)	56 (12.7)		0 (0.0)	
Tumor size (cm), mean ± SD		2.5 ± 0.9	2.6 ± 0.8	0.106	2.6 ± 1.0	0.570
Pathologic stage, n (%)	0	2 (2.4)	18 (4.1)	0.750	5 (3.0)	0.707
	I	38 (46.3)	194 (44.1)		66 (40.2)	
	II	42 (51.2)	228 (51.8)		93 (56.7)	
T classification, n (%)	T0	1 (1.2)	17 (3.9)	0.510	4 (2.4)	0.871
	T1	21 (25.6)	120 (27.3)		41 (25.0)	
	T2	55 (67.1)	267 (60.7)		106 (64.6)	
	T3	5 (6.1)	36 (8.0)		13 (7.9)	
N classification, n (%)	N0	43 (52.4)	216 (49.1)	0.202	68 (41.5)	0.135
	N1	37 (45.1)	189 (43.0)		73 (44.5)	
	N2	2 (2.4)	35 (18.0)		23 (14.0)	
Vessel resection, n (%)		5 (6.1)	130 (29.5)	< 0.001*	41 (25.0)	< 0.001*
Neoadjuvant therapy, n (%)		12 (14.6)	215 (48.9)	< 0.001*	53 (32.3)	0.005*
Adjuvant therapy, n (%)		72 (87.8)	403 (91.6)	0.208	130 (79.3)	0.141

*ASA* American Society of Anesthesiologists physical status classification system, *CA 19–9* Carbohydrate antigen 19–9, *R* resectable, *BR* borderline resectable, *LA* locally advanced, *M* metastatic

^*^P-values in statistical significance (P < 0.05)

### Survival analysis

#### Overall survival

**Fig. 3:**
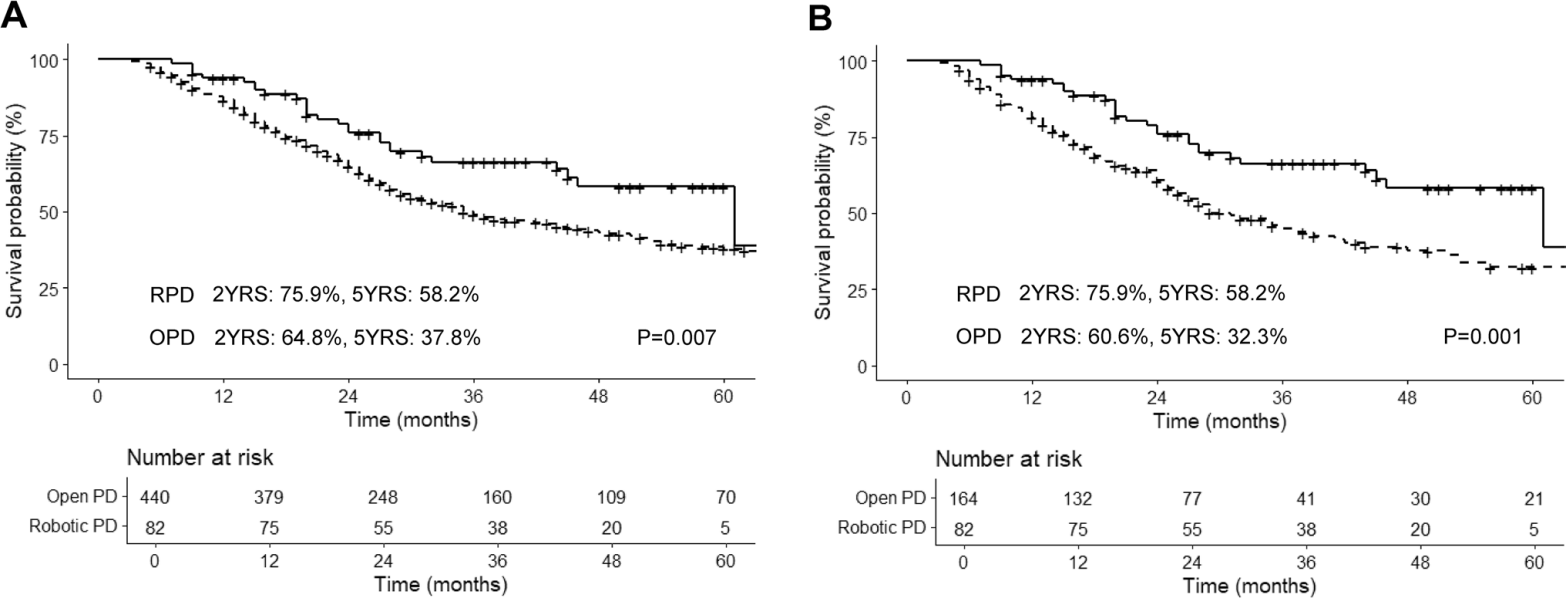
Kaplan–Meier curves of overall survival for RPD and OPD before PSM (**A**), after PSM (**B**). *PSM* propensity score matching, *RPD* robot-assisted pancreaticoduodenectomy, *OPD* open pancreaticoduodenectomy

OS between the RPD and OPD group showed significant difference (RPD versus OPD, median OS 61 months versus 35 months; 5-year OS 58.2% (95% CI, 46.5–72.8%) versus 37.8% (95% CI, 32.8–43.5%), P = 0.007) (Fig. 3A). After PSM (Fig. 3B), the RPD group still showed a significantly better OS than the OPD group (RPD versus OPD, median OS 61 months versus 31 months; 5-year OS 58.2% (95% CI, 46.5–72.8%) versus 32.3% (95% CI, 24.3–42.9%), P = 0.001) (Fig. 3B).

#### Disease-free survival

**Fig. 4:**
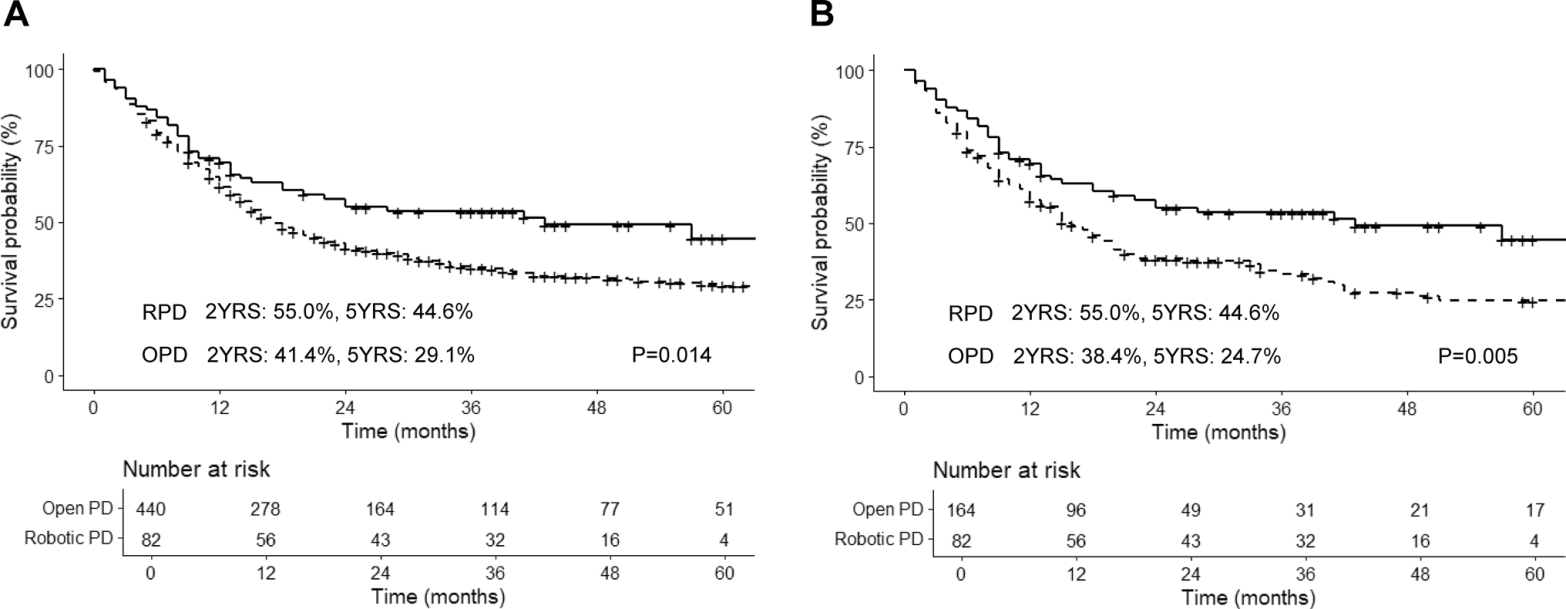
Kaplan–Meier curves of disease-free survival for RPD and OPD before PSM (**A**), after PSM (**B**). *RPD* robot-assisted pancreaticoduodenectomy, *OPD* open pancreaticoduodenectomy, *PSM* propensity score matching

The RPD group also demonstrated a favorable DFS rate, with a median survival of 43 months (5-year DFS rate of 44.6% (95% CI, 32.9–60.4%). The OPD group showed a significant difference from the RPD group before PSM (median DFS 17 months; 5-year DFS 29.1% (95% CI, 24.7–34.4%); P = 0.014) (Fig. 4A). Similarly, after PSM (Fig. 4B), the RPD group showed better DFS compared to the OPD group (RPD versus OPD, median DFS 43 months versus 16 months; 5-year DFS 44.6% (95% CI, 32.9–60.4%) versus 24.7% (95% CI, 17.8–34.2%), P = 0.005) (Fig. 4B).

#### Perioperative outcomes

Short-term perioperative outcomes were comparable between the RPD and OPD groups (Table 2). Before PSM, RPD had a longer operation time (RPD 331.9 ± 79.2 min versus OPD 295.6 ± 81.3 min, P < 0.001), and this difference persisted after PSM (331.9 ± 79.2 min versus 298.3 ± 86.2 min, P = 0.001). The number of retrieved lymph nodes was fewer in the RPD group than the OPD group before PSM (mean, RPD 20.0 ± 7.3 versus OPD 22.0 ± 10.6, P = 0.039), but this difference was no longer significant after PSM (RPD 20.0 ± 7.3 versus OPD 23.1 ± 11.5, P = 0.090). Blood loss and R0 resection rates were comparable between the two groups, both before and after PSM. Furthermore, the overall complication rate (RPD 41.5% versus OPD (before PSM) 41.6%, P = 1.000; (after PSM) 40.2%, P = 0.963), and severe complication rate, defined as Clavien-Dindo classification ≥ 3 (RPD 14.6% versus OPD (before PSM) 13.2%, P = 0.859; (after PSM) 11.6%, P = 0.634), showed no significant differences. This finding was consistent across major complications, including clinically relevant postoperative pancreatic fistula, delayed gastric emptying, post-pancreatectomy hemorrhage, biliary fistula, and wound complications.

**Table 2:** Perioperative outcomes of RPD and OPD, before and after propensity score matching

			Before PSM		After PSM	
Variables		RPD (n = 82)	OPD (n = 440)	P-value	OPD (n = 164)	P-value
Operation time (min), mean ± SD		331.9 ± 79.2	295.6 ± 81.3	< 0.001*	298.3 ± 86.2	0.001*
Blood loss (mL), mean ± SD		557.8 ± 587.2	587.4 ± 631.9	0.625	484.1 ± 488.6	0.761
Retrieved lymph node, mean ± SD		20.0 ± 7.3	22.0 ± 10.6	0.039*	23.1 ± 11.5	0.090
Resection margin, n (%)	R0	76 (92.7)	386 (87.7)	0.270	149 (90.9)	0.809
	R1	6 (7.3)	54 (12.3)		15 (9.1)	
Recurrence, n (%)		36 (43.9)	267 (60.7)	0.007*	102 (62.2)	0.010*
Overall complication, n (%)		34 (41.5)	183 (41.6)	1.000	66 (40.2)	0.963
Severe complication (CD ≥ 3), n (%)		12 (14.6)	58 (13.2)	0.859	19 (11.6)	0.634
CR-POPF, n (%)		5 (6.1)	20 (4.5)	0.763	6 (3.7)	0.725
DGE, n (%)		4 (4.9)	17 (3.9)	0.902	6 (3.7)	0.735
PPH, n (%)		2 (2.4)	7 (1.6)	0.937	2 (1.2)	0.603
Biliary fistula, n (%)		2 (2.4)	7 (1.6)	0.937	4 (2.4)	1.000
Wound, n (%)		0 (0.0)	14 (3.2)	0.260	3 (1.8)	0.553
Time to flatus (day)		3.8 ± 1.4	4.0 ± 1.6	0.441	4.2 ± 1.8	0.099
Time to drain removal (day)		7.4 ± 6.2	9.1 ± 5.4	0.013*	10.2 ± 7.2	< 0.001*
Time to no fluid infusion (day)		6.3 ± 2.9	8.3 ± 6.4	< 0.001*	9.0 ± 5.1	< 0.001*
Time to IV PCA removal (day)		4.5 ± 1.7	5.5 ± 17.7	0.260	4.7 ± 2.2	0.608
Hospital stays (day)		9.6 ± 7.0	13.2 ± 10.6	< 0.001*	14.2 ± 11.5	< 0.001*
Adjuvant therapy completion, n (%)		63 (87.5)	290 (78.0)	0.094	98 (75.4)	0.062
Adjuvant therapy cessation, n (%)		9 (12.5)	82 (22.0)	0.094	32 (24.6)	0.062
Patient tolerance		4 (5.6)	51 (13.7)	0.084	21 (16.2)	0.049*
Early disease progression		5 (6.9)	31 (8.3)	0.873	11 (8.5)	0.912
Op to adjuvant therapy (day)		46.7 ± 16.2	50.4 ± 34.9	0.171	49.1 ± 15.8	0.099

*CD* Clavien-Dindo classification grade, *CR-POPF* clinically relevant postoperative pancreatic fistula, *DGE* delayed gastric emptying, *PPH* postoperative pancreatic hemorrhage, *IV PCA* intravenous patient-controlled analgesia

^*^P-values in statistical significance (P < 0.05)

#### Postoperative functional recovery

For functional recovery indicators, the RPD showed shorter postoperative recovery time, in the time to drain removal (RPD 7.4 ± 6.2 days versus OPD (before PSM) 9.1 ± 5.4 days, P = 0.013; (after PSM) 10.2 ± 7.2 days, P < 0.001), the time to discontinuation of fluid infusion (RPD 6.3 ± 2.9 days versus OPD (before PSM) 8.3 ± 6.4 days, P < 0.001; (after PSM) 9.0 ± 5.1 days, P < 0.001, and length of hospital stay (RPD 9.6 ± 7.0 days versus OPD (before PSM) 13.2 ± 10.6 days, P < 0.001; (after PSM) 14.2 ± 11.5 days, P < 0.001), both before and after PSM.

#### Adjuvant therapy tolerance

After surgery, a comparable number of patients underwent adjuvant therapy in both groups (RPD 87.8% versus OPD (before PSM) 91.6%, P = 0.208; (after PSM) 79.3%, P = 0.141). In terms of the completion of adjuvant therapy, the RPD group had a completion rate of 87.5% compared with 75.4% in the OPD group after PSM (P = 0.062). The patients who could not complete adjuvant therapy were recategorized by the cause of cessation. A comparable proportion of patients with early disease progression during adjuvant therapy were converted to palliative settings (RPD 6.9% versus matched OPD 8.5%, P = 0.912). Notably, the number of patients who ceased adjuvant therapy due to deterioration in patient tolerance was estimated, and the RPD group showed significantly lower adjuvant therapy cessation than the matched OPD group (5.6% versus 16.2%, P = 0.049), despite no difference in the initial ASA status between the two groups. The time from surgery to initiation of adjuvant therapy was relatively short in both groups and showed no significant differences between the groups. These findings should affect the survival gap between RPD and OPD.

#### Prognostic factors for survival

Cox proportional hazards regression analysis was performed to identify prognostic factors for OS (Table 3). In the univariable analysis, elevated initial CA19-9 level (≥ 150 U/L) (HR 1.441 (95% CI 1.001–2.076, P = 0.049), positive nodal status (versus N0, HR 1.815 (95% CI 1.226–2.689, P = 0.003)), completion of adjuvant therapy (HR 0.381 (95% CI 0.242–0.601, P < 0.001)), and operation modality, whether robot-assisted or open (HR 0.503 (95% CI 0.328–0.771, P = 0.002) were associated with OS. In multivariate analysis, positive nodal status (versus N0, HR 2.003 (95% CI 1.221–3.286, P = 0.006)) and completion of adjuvant therapy (HR 0.415 (95% CI 0.261–0.660, P < 0.001)) were identified as significant prognostic factors for OS. The surgical modality was not significantly associated with OS in the multivariate analysis, with an HR of 0.750 (95% CI 0.461–1.220, P = 0.247).

**Table 3:** Cox proportional hazards regression analysis for overall survival

		Univariable analysis			Multivariable analysis		
Variables		HR	95% CI	P-value	HR	95% CI	P-value
Age (year)	≥ 70	1.267	0.860–1.865	0.231	–	–	–
Sex	Female	0.956	0.665–1.374	0.807	–	–	–
	Male	Reference					
Initial CA 19–9, U/L	≥ 150	1.441	1.001–2.076	0.049*	1.159	0.750–1.791	0.507
Clinical stage	LAPC	2.701	0.991–7.365	0.052	1.255	0.170–9.251	0.824
	R/BR	Reference					
T stage	T3,4 T1,2	1.369 Reference	0.690–2.717	0.370	–	–	–
N stage	N + N0	1.815 Reference	1.226–2.689	0.003*	2.003	1.221–3.286	0.006*
Resection margin	R1 R0	1.031 Reference	0.519–2.045	0.931	–	–	–
Harvested LNs number	≥ 15	1.552	0.958–2.515	0.074	1.601	0.868–2.952	0.132
Neoadjuvant therapy	Yes	0.886	0.555–1.413	0.611	–	–	–
Adjuvant completion	Yes	0.381	0.242–0.601	< 0.001*	0.415	0.261–0.660	< 0.001*
Operation modality	Robot Open	0.503 Reference	0.328–0.771	0.002*	0.750	0.461–1.220	0.247

*CA 19–9* Carbohydrate antigen 19–9, *LAPC* locally advanced pancreatic cancer, *R* resectable, *BR* borderline resectable, *LN* lymph node

^*^P-values in statistical significance (P < 0.05)

## Discussion

This study compared long-term oncological outcomes of RPD and OPD in patients diagnosed with pancreatic cancer. The RPD group demonstrated superior OS and DFS rates as compared to the open approach group for pancreatic cancer patients after stratification by age, sex, the ASA class, and clinical resectability. In addition, there were no significant differences between the two groups in terms of R0 resection, harvested LN numbers, and overall complication rates after PSM.

Our findings are consistent with those of previous studies that reported similar trends in survival outcomes between RPD and OPD. Wang SE et al. (2018) found a 2-year survival rate of 64.8% for RPD and 40.0% for OPD, while Shyr YM et al. (2021) reported a median survival of 15.3 months for RPD and 12.3 months for OPD in PDAC patients [18–20]. On the other hand, our study demonstrated generally better survival outcomes for both RPD and OPD groups compared to these reports. This discrepancy could be attributed to the more recent patient population in this study, the exclusion of advanced stages in the analysis, and the increased use of neoadjuvant and adjuvant chemotherapy protocols, which collectively improved the pancreatic cancer survival rates over time. Using the National Cancer Database (NCDB), Nassour et al. (2021) analyzed the outcomes of RPD and OPD in patients with pancreatic cancer who underwent neoadjuvant chemotherapy. They also reported similar rates of the positive margin status (RPD 21% versus OPD 16%), and a shorter length of hospital stay in RPD group (RPD 8 ± 5 versus OPD 10 ± 7, P < 0.001), findings that are consistent with our study. However, in survival analysis, they found no significant difference in the OS rates (median OS: RPD 25.6 months versus OPD 27.5 months, P = 0.879). This discrepancy should be interpreted with caution, considering the nature of the NCDB data, which aggregates information from diverse facilities. Notably, the proportion of patients treated at large-volume facilities, including academic/research programs and integrated network cancer programs, differed between the RPD (95%) and OPD (85%) groups. These variations in institutional composition may have influenced both surgical outcomes and OS results [21].

In the Cox regression analysis for OS, the surgical modality was not a relevant factor (HR 0.750, 95% CI 0.461–1.220). This implies that the robotic technique itself is not the actual effective variable for prognosis; rather, the sequentially affected variables are the true determinants of survival. Another independent prognostic factor for survival was the completion of adjuvant therapy (HR 0.415, 95% CI 0.261–0.660). The rate of discontinuation of adjuvant therapy owing to a deteriorating condition was lower in the RPD group (5.6% vs. 16.2%, P = 0.049). DePeralta et al. [22] reported that completion of adjuvant therapy was an independent prognostic factor for both recurrence-free survival (HR 0.52, 95% CI 0.359–0.755) and overall survival (HR 0.410, 95% CI 0.274–0.614) in pancreatic ductal adenocarcinoma, which is comparable to our analysis [22].

The major reasons for failure to initiate or continue adjuvant therapy were drug toxicity and poor functional status of the patients. In the RPD group, 4 patients did not initiate or discontinued adjuvant chemotherapy, due to poor performance status (n = 2) and patient preference (n = 2). In the OPD group, 52 patients withheld adjuvant chemotherapy for similar reasons, including poor performance status (n = 32) and patient preference (n = 20). Adverse effects leading to discontinuation of adjuvant therapy were more frequently reported in the OPD group.

Indicators of recovery from surgery, such as the time to drain removal, fluid infusion discontinuation, and hospital stay, were significantly shorter in the RPD group. Recently, two randomized trials comparing the short-term outcomes of RPD and OPD reported conflicting results [23, 24]. The trend of our study was consistent with findings from Liu et al. (2024). They reported that the RPD group had a shorter hospital stay (11.0 [9.0–20.0] versus 14.0 [12.0–21.5] days) and a faster postoperative recovery, including earlier time to first flatus (2.0 [1.0–2.0] versus 2.0 [2.0–3.0] days), and a shorter duration of analgesic use (6.0 [5.0–8.0] versus 8.0 [6.0–10.0] days) [23]. These may denote that robotic surgery may be associated with reduced surgical stress or mitigate immediate postoperative decline, potentially enabling patients to achieve tolerable conditions for adjuvant chemotherapy more quickly. The time to initiation of adjuvant therapy after surgery did not differ significantly between the two groups (RPD 46.7 vs. OPD 47.8 days, P = 0.878). Notably, previous studies have reported that the delayed initiation of adjuvant therapy is not associated with OS in patients with pancreatic cancer [25, 26]. The actual assessment for progression of postoperative physical decline should be further investigated in future studies to validate this hypothesis.

Conversely, another recent randomized study Klots et al. [24] reported that the RPD group was associated with a longer hospital stay and a higher incidence of major complications than the OPD group. However, they also observed a relatively high rate of overall complications (58.6%) and a clinically relevant POPF incidence (37.9%) in the RPD group [24]. Given these findings, it is important to consider the possible impact of the learning curve in the RPD when interpreting these results.

Besides, possible bias in initial patient selection should be carefully considered. Surgeons often tend to select patients with better health status and fewer comorbidities for robotic surgeries. Although the surgical groups were matched for sex, age, ASA class, and resectability, excluding stage III and IV cancers in this study, it remains challenging to retrospectively assess the baseline performance status at the time of surgery to evaluate bias. Also, the rate of combined vessel resection was varied between two groups, implying possible discrepancy in the clinical resectability between two groups. In addition, the RPD and OPD groups showed different proportions of neoadjuvant therapy (14.6% versus 48.9%, after matching 32.3%). This finding can affect the survival gap between groups. However, additional subgroup analysis of the neoadjuvant and non-neoadjuvant subgroups showed no specific differences in long-term survival outcomes between the RPD and OPD groups. As the proportion of RPD candidates receiving neoadjuvant therapy gradually increases over time, future studies should include more balanced population groups for analysis.

Another potential source of bias is the difference in perioperative management strategies. In South Korea, patients who undergo robotic surgery are often of higher socioeconomic status due to the substantial cost difference between robotic and conventional surgical approaches. This socioeconomic disparity may influence postoperative recovery and access to adjuvant treatment.

Furthermore, the time distribution between the two groups should be taken into account. A higher proportion of patients in the RPD group underwent surgery in more recent years (56.1% vs. 39.0%), which may reflect evolving treatment strategies, including increased use of neoadjuvant or adjuvant chemotherapy. These temporal differences could have contributed to improved survival outcomes in the RPD group.A limitation of our study is the potential patient selection bias, as the assignment of robotic surgery was influenced by the surgeon or the patients themselves, as discussed above. Additionally, the exclusion of patients with advanced-stage disease and intraoperative open conversion to minimize bias restricts the comparison of oncologic efficacy to early stage and clinically resectable pancreatic cancer. The limitations of this retrospective study should be considered. These limitations should be considered further and analyzed in prospective settings in future studies. 

A potential limitation is residual confounding from unmatched factors such as preoperative CA19-9 and nodal stage. These were not included in the matching process, as no significant between-group differences were observed after matching. However, their clinical importance is acknowledged, and future studies should consider incorporating such variables for more robust adjustment.

## Conclusion

In conclusion, this study suggests that RPD is a feasible alternative to OPD, offering certain short-term postoperative advantages without compromising long-term oncological outcomes in pancreatic cancer. The observed trend towards better outcomes in the RPD group, coupled with its higher adjuvant therapy completion rate, underscores the potential oncological benefits of this minimally invasive approach. Further prospective multicenter studies with balanced populations are needed to validate these findings and optimize patient selection criteria for robot-assisted pancreatic surgery for pancreatic cancer.

## Data Availability

All data analyzed during this study are included in this article (and its supplementary material) as references to published articles or are available from the corresponding authors on reasonable request.
